# The HSV-1 Transcription Factor ICP4 Confers Liquid-Like Properties to Viral Replication Compartments

**DOI:** 10.3390/ijms22094447

**Published:** 2021-04-24

**Authors:** Michael Seyffert, Fanny Georgi, Kurt Tobler, Laurent Bourqui, Michela Anfossi, Kevin Michaelsen, Bernd Vogt, Urs F. Greber, Cornel Fraefel

**Affiliations:** 1 Institute of Virology, University of Zurich, CH-8057 Zurich, Switzerland; kurt.tobler@uzh.ch (K.T.); michela.anfossi@edu.unito.it (M.A.); kevin.michaelsen@uzh.ch (K.M.); bernd.vogt@uzh.ch (B.V.); cornel.fraefel@uzh.ch (C.F.); 2 Department of Molecular Life Sciences, University of Zurich, CH-8057 Zurich, Switzerland; fanny.georgi@uzh.ch (F.G.); urs.greber@uzh.ch (U.F.G.); 3 Institute of Anatomy, University of Zurich, CH-8057 Zurich, Switzerland; laurent.bourqui@anatomy.uzh.ch; 4 Molecular, Cellular and Developmental Biology (MCD), Center for Integrative Biology (CBI), University of Toulouse, National Center for Scientific Research (CNRS), 31055 Toulouse, France

**Keywords:** Herpes Simplex Virus Type-1, viral transcription factor, ICP4, intrinsically disordered proteins, liquid–liquid phase separation, viral replication compartments

## Abstract

Herpes Simplex Virus Type-1 (HSV-1) forms progeny in the nucleus within distinct membrane-less inclusions, the viral replication compartments (VRCs), where viral gene expression, DNA replication, and packaging occur. The way in which the VRCs maintain spatial integrity remains unresolved. Here, we demonstrate that the essential viral transcription factor ICP4 is an intrinsically disordered protein (IDP) capable of driving protein condensation and liquid–liquid phase separation (LLPS) in transfected cells. Particularly, ICP4 forms nuclear liquid-like condensates in a dose- and time-dependent manner. Fluorescence recovery after photobleaching (FRAP) assays revealed rapid exchange rates of EYFP-ICP4 between phase-separated condensates and the surroundings, akin to other viral IDPs that drive LLPS. Likewise, HSV-1 VRCs revealed by EYFP-tagged ICP4 retained their liquid-like nature, suggesting that they are phase-separated condensates. Individual VRCs homotypically fused when reaching close proximity and grew over the course of infection. Together, the results of this study demonstrate that the HSV-1 transcription factor ICP4 has characteristics of a viral IDP, forms condensates in the cell nucleus by LLPS, and can be used as a proxy for HSV-1 VRCs with characteristics of liquid–liquid phase-separated condensates.

## 1. Introduction

Replication of DNA viruses, such as herpesvirus, parvovirus, papillomavirus, or adenovirus, occurs in distinct inclusions within the nucleus. These inclusions are called viral replication compartments (VRCs) and constitute membrane-less, spherical domains where viral transcription, viral DNA (vDNA) replication, virion assembly, and genome packaging occur (reviewed in [1,2,3,4]).

Differently from DNA viruses, most RNA viruses replicate in the cytoplasm of susceptible cells, where they form membrane-bound or membrane-less viral assemblies. Such membrane-less viral compartments in the cytoplasm are called viral factories or viroplasms and, similarly to DNA viruses, constitute distinct domains where genome replication and packaging occur (reviewed in [5]). Recently, two independent studies reported that the viral factories of vesicular stomatitis virus (VSV) and rabies virus (RabV) share biophysical properties of liquid–liquid phase-separated condensates in the cell, and have liquid-like characteristics, such as fusion upon close contact or rapid growth over time [6,7]. Furthermore, several viral factors that directly or indirectly account for the liquid-like nature of these viral factories were identified. Apparently, the formation of VSV factories is driven by the condensation of the viral phosphoprotein (P) together with the nucleocapsid protein (N) and the multifunctional large protein (L) via liquid–liquid phase separation (LLPS) [6]. RabV factories, on the other hand, are formed by the N and P proteins alone [7]. Importantly, the P proteins of both viruses comprise large intrinsically disordered regions (IDRs) capable of establishing weak hydrophobic interactions with other IDRs and RNA. It was demonstrated that this characteristic feature of intrinsically disordered proteins (IDPs) is a major driver of LLPS and the subsequent condensation of macromolecules in the cell [8,9]. In addition, RNA-binding proteins harboring classical RNA-binding motifs, such as the K homology (KH) or RNA recognition motif (RRM) domains, play an important role in the formation and maintenance of phase-separated condensates [10]. In fact, VSV N and L proteins can bind RNA through RNA-binding domains and hence may contribute to the formation of phase-separated viral factories by recruiting cellular or viral RNA.

In accordance, it is tempting to speculate that nuclear VRCs of DNA viruses such as HSV-1 are phase-separated condensates as well [2,3,11]. Several characteristics of HSV-1 VRCs support this hypothesis, including their spherical shape early in infection, the capability to fuse upon physical contact, and the fact that many HSV-1 proteins are predicted to be highly disordered [12,13,14,15]. Here, we provide experimental data demonstrating that the HSV-1 transcription factor (TF) ICP4 is a viral IDP capable of driving protein condensation and that ICP4 confers its liquid-like properties to VRCs.

## 2. Results

### 2.1. The HSV-1 Transcription Factor ICP4 Is a Viral IDP That Drives the Formation of Nuclear Condensates

**Figure 1:**
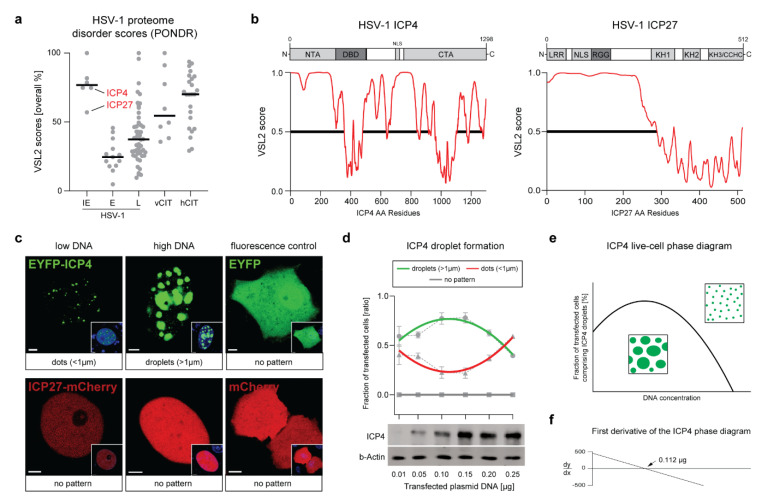
The HSV-1 transcription factor ICP4 is a viral IDP that drives the formation of nuclear condensates. (**a**) HSV-1 proteome screening for intrinsically disordered proteins (IDPs). A total of 71 HSV-1 proteins were screened for the presence of intrinsically disordered regions (IDRs) using the VSL2 algorithm from the online platform PONDR (see also Table S2). VSL2 scores of each protein are plotted as overall percentage disorder. The HSV-1 proteins are separated according to their kinetic expression profile: immediate–early (IE), early (E), and late (L) proteins. A selection of cited viral (vCIT) and human (hCIT) IDPs that phase-separate are shown as a reference (see also Tables S1 and S3). The two essential IE proteins, ICP4 and ICP27, are highlighted. Black bars represent the mean overall percentage VSL2 scores. (**b**) The VSL2 disorder score profiles of ICP4 and ICP27. The relevant protein domains of both proteins are shown above the corresponding VSL2 profiles. The IDRs are highlighted with horizontal black lines. Abbreviations: NTA, N-terminal activation domain: CTA, C-terminal activation domain; DBD, DNA-binding domain; NLS, nuclear localization signal; LRR, Leucine-rich region; RGG, Arg-Gly-Gly box; KH1/2/3, K-Homology domains 1/2/3; CCHC, Cys/Cys/His/Cys Zn-finger domain; (**c**) ICP4 but not ICP27 forms condensates in the nuclei of transfected cells. Vero cells were transfected with either pIE3-EYFPICP4 or mCherry2-ICP27 using two different plasmid DNA concentrations: 0.01 (low DNA) and 0.15 µg (high DNA). At 16 hpt, the cells were counterstained with Hoechst 33342 and processed for live cell imaging using a CLSM. Single z-scans of representative cell nuclei are shown. The plasmids expressing EYFP or mCherry (0.15 µg each) were used as negative controls. Scale bars: 3µm (ICP4 and ICP27), 5 µm (EYFP), and 10µm (mCherry); (**d**) The formation of ICP4 droplets is dose-dependent. Vero cells were transfected with different amounts of pIE3-EYFPICP4 (0.01, 0.05, 0.1, 0.15, 0.2, and 0.25 µg). At 16 hpt, the fraction of cells comprising ICP4 droplets, dots, or no pattern was determined for each transfection (dashed grey line). The graph represents data from three independent experiments where 50–100 transfected cells were counted. Regression curves for each category (green, red, or grey lines) were evaluated using Prism (GraphPad). Western blot analysis was performed in order to confirm the different ICP4 protein levels in transfected cells. The EYFP-ICP4 fusion protein was detected with a monoclonal antibody against ICP4 (ICP4). The beta-actin protein levels served as a loading control (b-Actin); (**e**) The droplets regression curve from panel (**d**) was used to derive the complete ICP4 live-cell phase diagram. The region below the curve represents the conditions that favor de-mixing of ICP4 into droplets. (**f**) The first derivative of the ICP4 phase diagram revealed that the summit of the curve is defined at the DNA amount 0.112 µg.

The formation and maintenance of liquid-like viral factories from RNA viruses in the cytoplasm is promoted by viral IDPs (vIDPs) that drive protein condensation via LLPS, such as the phosphoproteins (P) of RabV or VSV (Table S1) [6,7]. Thus, it is feasible that also DNA virus VRCs have liquid-like properties and that their formation and maintenance is driven by vIDPs that can phase-separate. In order to interrogate the liquid-like properties of HSV-1 VRCs, we sought to identify and characterize candidate vIDPs that can drive LLPS. Here, we used the VSL2 algorithm provided by the online platform PONDR to evaluate the overall percentage disorder of 71 HSV-1 proteins in silico and compared them with known viral and human IDPs that have been shown to phase-separate in cells (Figure 1a and Tables S1–S3). Confirming the previous findings of McSwiggen et al. [13], we found that all HSV-1 IE proteins are generally highly disordered, rendering this class of viral proteins ideal candidate vIDPs. In order to narrow down the list of candidate HSV-1 IDPs, we considered hallmarks of phase-separating vIDPs, including the capability to bind nucleic acids (DNA or RNA), the localization within VRCs, and the essentiality for virus replication (Table S2). Using these criteria, we identified two viral factors as potential drivers of LLPS: the major viral TF ICP4 and the viral RNA-processing factor ICP27 (Figure 1a,b and Table S2). Either protein is essential for virus replication, can bind nucleic acids through distinct DNA or RNA-binding motifs, and comprises large IDRs that include a compositional bias of highly charged aromatic amino acids (AAs) capable of driving LLPS via pi–pi stacking (Figure 1b and Figure S1a,b). Because ICP4 and ICP27 proteins both localize in HSV-1 VRCs [16,17,18] and are expressed already before vDNA replication occurs [19,20], they are ideal candidate vIDPs.

When transiently expressed in cell culture, cellular and viral IDPs that drive LLPS are prone to spontaneously de-mix and form distinct condensates in a concentration-dependent manner. We tested the ability of ICP4 and ICP27 to induce the formation of phase-separated condensates in the cell and assessed whether this process is concentration-dependent. Vero cells were transfected with a low (0.01 µg) or a high (0.25 µg) amount of plasmid DNA expressing either ICP4, which is N-terminally fused to EYFP (pIE3-EYFPICP4), or ICP27, which is C-terminally fused to mCherry (mCherry2-ICP27). We found that at 16 h post transfection (hpt), ICP4 predominantly formed small distinct puncta (dots) in the nucleus when a low concentration of plasmid DNA was used (Figure 1c). In contrast, when a high amount of plasmid DNA was transfected, the majority of transfected cells exhibited larger ICP4 droplet-like condensates in the nucleoplasm (Figure 1c). In order to distinguish these two patterns in subsequent experiments, we determined ICP4 condensates with a diameter of <1 µm as dots and condensates with a diameter of >1 µm as droplets.

On the other hand, ICP27 did not induce the formation of any kind of dots or droplet-like structures when expressed alone (Figure 1c). Of note, early in infection, ICP27 regulates the transcriptional activity of ICP4 by controlling the degree of ICP4 phosphorylation, which may play a role in the ability of ICP4 to phase-separate [21]. Therefore, we hypothesized that ICP27 may co-localize with ICP4 droplets in the nucleus via ICP4-induced LLPS. To test this possibility, we co-transfected cells with both plasmids and found that ICP27 did not co-localize with any ICP4-induced dots or droplets (Figure S1c). Moreover, the ability of ICP4 to form droplets was not altered in the presence of ICP27, suggesting that ICP4 phase separation is a process independent of ICP27.

Concentration-dependent de-mixing is a hallmark of IDPs and can be demonstrated by phase diagrams in vitro and in cell cultures. To test whether ICP4 phase separation follows the norms of a characteristic phase diagram in cell culture, we transfected different amounts of pIE3-EYFPICP4 into Vero cells and evaluated the number of transfected cells exhibiting ICP4 droplets, dots, or no patterns at 16 hpt (Figure 1d). The number of cells harboring ICP4 droplets changed depending on the amount of transfected plasmid DNA, resulting in a parabolic curve typical for phase diagrams of phase-separating IDPs. Western blot analysis confirmed the different ICP4 protein levels in transfected cells (Figure 1d, lower panel). Following interpolation of the phase diagram (Figure 1e), we determined that 0.112 µg of transfected plasmid DNA resulted in the highest number of cells with ICP4 droplets when using our transfection setting, as seen in the plots representing the phase diagram and its first-order derivative (Figure 1f).

These data demonstrate that HSV-1 ICP4 is a vIDP that can drive the formation of condensates in transfected cells, possibly via LLPS. In order to confirm these results, we next aimed at determining whether ICP4 condensates have liquid-like properties.

### 2.2. ICP4 Condensates Have Liquid-Like Properties

**Figure 2:**
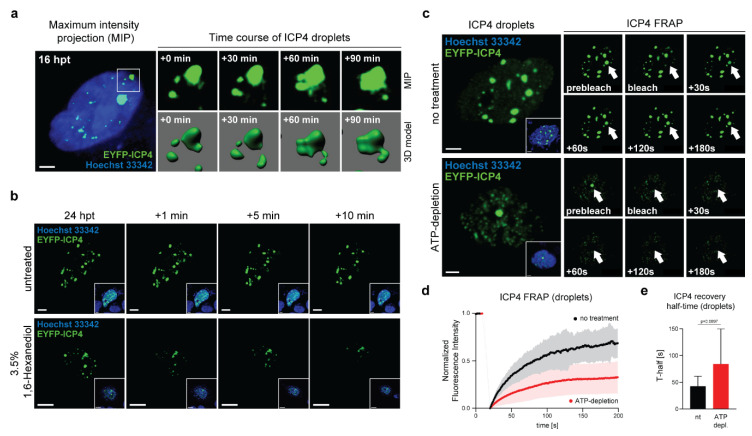
ICP4 condensates have liquid-like properties. (**a**) 3D live-cell imaging of ICP4 condensates. Vero cells were transfected with 0.3 µg of pIE3-EYFPICP4. At 16 hpt, the cells were counterstained with Hoechst 33342 and monitored for 3 h using a spinning disk CLSM (see also Movies S1a,b). A representative cell nucleus displaying ICP4 droplets was selected for 3D modeling using the Imaris software (Bitplane). ICP4 droplet fusion events are indicated in maximum intensity projection (MIP) and rendered 3D images. Scale bar: 10 µm. (**b**) ICP4 condensate integrity is compromised by 1,6-Hexanediol. Vero cells were transfected with 0.15 µg of pIE3-EYFPICP4 and counterstained with Hoechst 33342 at 24 hpt followed by live-cell microscopy using CLSM. The cells were treated with 3.5% 1,6-Hexanediol and fluorescence images were taken at indicated times. Untreated cells were used as a negative control. Scale bars: 10 µm (1,6-Hexanediol) and 3 µm (untreated). (**c**) ICP4 droplets exhibit rapid FRAP kinetics. Vero cells were transfected with 0.15 µg of pIE3-EYFPICP4 and counterstained with Hoechst 33342 at 24 hpt (inset images) followed by FRAP analysis using CLSM. A single ICP4 droplet was bleached (white arrow) and then monitored for 3 min capturing an image every second (see also Movie S2). ATP depletion was performed 2 h before the FRAP experiment (see also Movie S3). Scale bar: 5 µm. (**d**) Quantification and analysis of FRAP experiments. For FRAP analysis, 6–12 individual ICP4 droplets were used. The raw FRAP data were normalized with easyFRAP and analyzed with Prism (GraphPad). The normalized fluorescence intensity is plotted over time (s). (**e**) ICP4 recovery half-times (T-half) were evaluated using the FRAP data from panel (2d) with easyFRAP. Bar plots represent mean values with standard deviations (SDs). Statistical significance was determined using a Welch’s *t*-test.

The properties of liquid-like condensates in the cell include spatio-temporal features, such as fusion upon physical contact, growth over time, or a spherical shape. In order to investigate the liquid-like behavior and the spatio-temporal dynamics of ICP4 condensates, we performed 3D live-cell imaging using a spinning-disc confocal laser scanning microscope (CLSM). Vero cells were transfected with pIE3-EYFPICP4, stained with Hoechst 33342 at 16 hpt [22], and subjected to live-cell imaging. Z-stacks of the specimen were captured every 10 min for 3 h. Maximum-intensity projections (MIP) and the rendered 3D images of a representative nucleus displaying EYFP-ICP4 dots and droplets are shown in Figure 2a (see also Movies S1a,b). During the time course, smaller ICP4 droplets tended to freely move within the nucleus and undergo fusion, resulting in the formation of droplets.

The aliphatic alcohol 1,6-Hexanediol interferes with weak hydrophobic protein–protein and protein–RNA interactions and can dissolve a variety of cellular phase-separated condensates [23]. We tested the effect of 1,6-Hexanediol on EYFP-ICP4 condensates and found that they were heavily compromised (Figure 2b). Compared to the untreated control, ICP4 condensates started to dissolve after 1 min of treatment and almost completely disappeared after 10 min.

Another characteristic property of phase-separated condensates is the rapid diffusion kinetics of the phase-separated molecules. We used fluorescence recovery after photo-bleaching (FRAP) experiments to assess the diffusion dynamics of ICP4 in ICP4-induced droplets established by transfecting pIE3-EYFPICP4 and analyses at 24 hpt (Figure 2c–e and Movie S2). We found rapid ICP4 diffusion dynamics in ICP4 droplets where up to 75% of the initial normalized fluorescence intensity (NFI) was restored within 3 min of recovery at a mean recovery half-time (T-half) of 42.4 s (Figure 2d,e). We next tested whether ATP depletion had an impact on the ICP4 diffusion dynamics in ICP4 droplets. ATP acts a hydrotrope and promotes fluidity in phase-separated condensates. Depletion of cellular ATP alters the FRAP dynamics of phase-separating IDPs [24,25]. ICP4 droplets in Vero cells were established as described above and ATP depletion was accomplished with the ATP synthase inhibitor Oligomycin A for 30 min, followed by FRAP analysis (Figure 2c–e and Movie S3). We found that the fluorescence recovery of ICP4 in ATP-depleted cells was significantly decreased and reached a maximum of approximately 25% NFI after 3 min and the average T-half was increased to 84.01 s (Figure 2d,e).

Altogether, these results indicate that ICP4 droplets have liquid-like properties typical of proteins that can undergo LLPS.

### 2.3. The ICP4 NTA and DBD Control the Protein Phase Behavior

**Figure 3:**
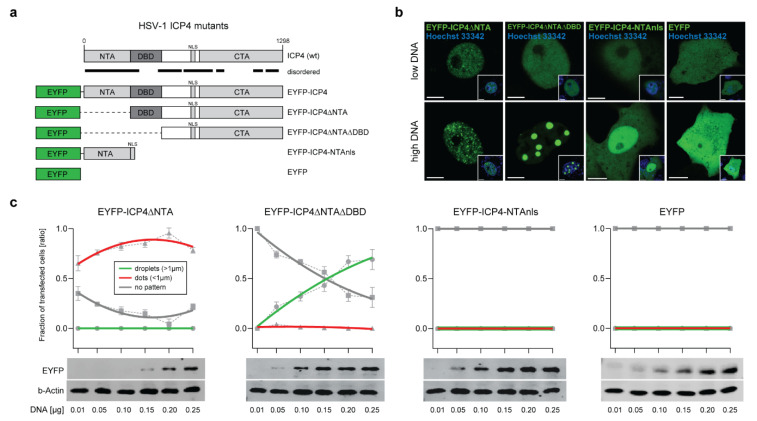
The ICP4 NTA and DBD control the protein phase behavior. (**a**) Schematic representation of the mutant HSV-1 ICP4 constructs used in this study. The plasmid expressing EYFP-ICP4 was used as a positive control and the plasmid expressing EYFP alone was used as a negative control. Disordered regions are indicated with black lines. (**b**) Phase behavior of the mutant ICP4 constructs. Vero cells were transfected with either 0.01 µg (low DNA) or 0.15 µg (high DNA) plasmid DNA expressing the ICP4 constructs described in panel 3a. The cells were counterstained with Hoechst 33342 at 16 hpt (small inset) and subjected to live-cell imaging using CLSM. Representative images for each construct are shown. Scale bars: 5 µm (EYFP-ICP4 constructs) and 10 µm (EYFP). (**c**) Live-cell phase diagrams of the mutant ICP4 constructs. The generation of the protein phase diagrams was performed as described for the EYFP-ICP4 construct (see Figure 1d). The Western blot analysis to detect all mutant ICP4 constructs was performed using a monoclonal antibody against GFP (JL-8) that is capable of detecting EYFP (EYFP). The beta-actin protein levels served as a loading control (b-Actin).

In order to better understand the liquid-like behavior of ICP4, we sought to identify the protein domains that drive the formation of ICP4 condensates. Protein IDRs and RNA- or DNA-binding domains can control the liquid-like behavior of macromolecules in the cell [10]. We tested whether the ICP4 DBD, the CTA, or the NTA were required for the formation of ICP4 droplets in transfected cells. A set of mutant pIE3-EYFPICP4 constructs were generated lacking either the NTA (EYFP-ICP4∆NTA), the NTA and the DBD (EYFP-ICP4∆NTA∆DBD), or the CTA and the DBD (EYFP-ICP4-NTAnls) (Figure 3a). We then evaluated the phase behavior of these mutant EYFP-ICP4 proteins in transfected Vero cells (Figure 3b,c). First, we compared cells that were transfected with a low (0.01 µg) or a high (0.15 µg) plasmid DNA amount using CLSM. No mutant EYFP-ICP4 construct formed any kind of dots or droplets in cells transfected with a low amount of plasmid DNA, except EYFP-ICP4∆NTA, which sporadically formed a small number of dots in certain nuclei (Figure 3b). In cells transfected with a high amount of plasmid DNA, we observed a large number of dots for EYFP-ICP4∆NTA, but no droplets, whereas in cells transfected with EYFP-ICP4∆NTA∆DBD, we found large droplets in more than half of transfected cells, but no dots. The EYFP-ICP4-NTAnls protein did not form any dots or droplets at either transfection condition.

Since EYFP-ICP4 protein condensation is dose-dependent and follows the norms of typical IDP phase diagrams, we examined whether the phase behavior of the mutant EYFP-ICP4 constructs is also dose-dependent. For this purpose, we evaluated the number of nuclei displaying either dots, droplets, or no particular EYFP-ICP4 features (no pattern) for each construct in cells transfected with different amounts of plasmid DNA (Figure 3c). We found that EYFP-ICP4∆NTA did not form droplets at any transfection condition, but the number of transfected cells with dots increased in proportion with the plasmid DNA amount that was transfected, while the number of cells with no pattern decreased accordingly. Interestingly, EYFP-ICP4∆NTA∆DBD formed droplets exclusively and the number of cells with droplets continuously increased the more plasmid DNA was transfected, while no cells displayed any dots. On the contrary, neither dots nor droplets were found in any transfection with the EYFP-ICP4-NTAnls construct. The plasmid pIE3-EYFP expressing EYFP alone was used as a negative control (Figure 3b,c). In order to control for the protein expression levels of the different constructs, we performed a Western blot analysis of cells transfected with different plasmid DNA amounts (Figure 3c, lower panels). As expected, all constructs showed a comparable gradient of detected proteins according to the amount of transfected plasmid DNA.

These results demonstrate that the NTA plays a pivotal role in controlling ICP4 condensate features but does not drive the formation of ICP4 condensates per se, whereas the CTA or the linker region between the CTA and the DBD possibly are the main drivers of ICP4 condensation. Similar to the NTA, the DBD is most likely involved in controlling protein condensation.

### 2.4. ICP4 Retains Its Liquid-Like Properties in HSV-1 VRCs

**Figure 4:**
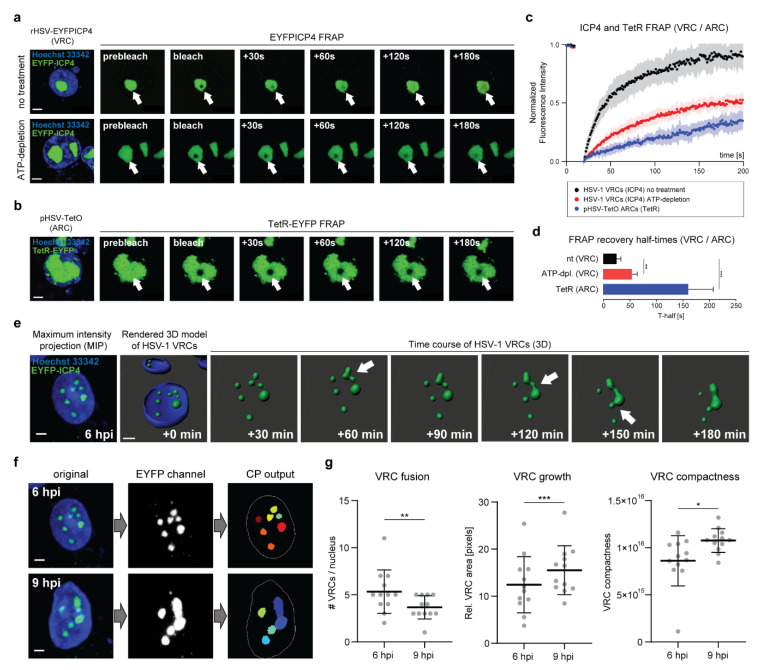
ICP4 retains its liquid-like properties in HSV-1 VRCs. (**a**) ICP4 exhibits rapid diffusion kinetics within HSV-1 VRCs. Vero cells were infected with rHSV-EYFPICP4 at a multiplicity of infection (MOI) of 1. At 12 hpi, the cells were counterstained with Hoechst 33342 and processed for FRAP using CLSM (SP8 Falcon, Leica). A circular area of 1 µm in diameter was bleached within the HSV-1 VRC using 10 iterations of 100% laser power. The bleached area was then monitored for 3 min and an image was captured every second (see also Movie S4). ATP depletion was performed at 10 hpi for 2 h and FRAP was performed as described above (see also Movie S5). Scale bars: 5 µm. (**b**) FRAP kinetics of HSV-1 amplicon DNA within amplicon replication compartments (ARCs). Vero cells were transfected with 0.5 µg of pHSV-TetO and 0.01 µg of pSV2-EYFP-TetR. At 16 hpt, the cells were infected with wt HSV-1 at a MOI of 2. FRAP experiments were performed at 12 hpi and analyzed as described in panel (4a) (see also Movie S6). (**c**) Quantification of the FRAP experiments. For FRAP analysis, 6–12 individual VRCs were used. The raw FRAP data were normalized with easyFRAP and analyzed with Prism (GraphPad). The normalized fluorescence intensity is plotted over time (s). (**d**) ICP4 recovery half-times (T-half) were evaluated with the FRAP data from panel (4c) using easyFRAP. Bar plots represent mean values with SDs. Statistical significance was determined using a Welch’s *t*-test; *p* < 0.005 (**) and *p* < 0.0005 (***). (**e**) 3D live-cell imaging of Vero cells infected with rHSV-EYFPICP4 (MOI 1). At 6 hpi, the nuclei were counterstained with Hoechst 33342 and processed for live imaging with a spinning disk confocal laser scanning microscope (CLSM). Z-stacks of the specimen were taken every 10 min for 3 h. A representative z-stack of an infected nucleus at 6 hpi is shown as a maximum-intensity projection (MIP) (left) and used for time-course 3D imaging using the Imaris software (Bitplane). Fusion events during the course of infection are indicated with white arrows. Images were taken from Movie S7 every 30 min; Scale bar: 5 µm. (**f**) Cell Profiler (CP) processing procedure for the quantification of the HSV-1 VRC phase behavior. MIP images of the representative cell nucleus from panel (4e) are shown as an example at 6 and 9 hpi. Scale bar: 5 µm. (**g**) Quantification of the HSV-1 VRCs phase behavior. A total of 12 infected cell nuclei were processed using the customized CP pipeline from panel (**f**). The number of VRCs per nucleus, the relative VRC size, and compactness were assessed at 6 and 9 hpi for each candidate nucleus. Horizontal bars represent mean values with error bars showing SD values. Statistical significance was determined with a Welch’s *t*-test; *p* < 0.05 (*), *p* < 0.005 (**), and *p* < 0.0005 (***).

We next tested whether ICP4 exhibits its liquid-like properties, such as the rapid diffusion dynamics, also within HSV-1 VRCs. To assess the ICP4 diffusion kinetics in HSV-1 VRCs, we performed FRAP experiments in Vero cells infected with rHSV-EYFPICP4, a recombinant HSV-1 expressing ICP4 N-terminally fused to EYFP (Figure 4a–d). The resulting VRCs in infected cells can be readily identified since, naturally, ICP4 is exclusively located in VRCs [26,27]. This setup allowed us to measure ICP4 diffusion directly in VRCs. We found that ICP4 FRAP kinetics were again rapid and fluorescence recovery was almost complete within 2–3 min (Figure 4a,c,d and Movie S4). ATP depletion also impacted the ICP4 diffusion dynamics in HSV-1 VRCs, as shown by Oligomycin A treatment and FRAP analysis, which indicated a 50% reduction in the maximum recovery and a two-fold increase in the mean T-half compared to untreated cells (Figure 4d).

Given the fact that ICP4 binds to the HSV-1 DNA in VRCs, it is plausible that the diffusion of the viral DNA rather than ICP4 itself accounts for the rapid ICP4 FRAP kinetics. Indeed, heterochromatin of eukaryotic cells is phase-separated from euchromatin by the heterochromatin protein 1 (HP1) via LLPS [28,29]. Similarly, the HSV-1 DNA might be phase-separated from cellular chromatin via LLPS, akin to heterochromatin. To test whether HSV-1 DNA is able to freely diffuse within VRCs, we utilized an HSV-1 amplicon system, which allows fluorescent labeling of the vDNA and, hence, the corresponding VRCs in live cells [30,31,32]. Vero cells were transfected with pHSV-TetO, an amplicon plasmid comprising both the HSV-1 origin of DNA replication (oriS) and a cassette comprising a series of 35 sequentially arranged tetracycline operon (TetO) sequences. Together with the HSV-1 amplicon, we co-transfected pSV2-TetR-EYFP, a plasmid expressing the tetracycline repressor (TetR) fused to EYFP. At 16 hpt, transfected cells were infected with wild-type (wt) HSV-1 in order to provide the viral proteins necessary for oriS-dependent DNA replication. In cells that were successfully transfected and infected, the replication of the HSV-1 amplicon DNA plasmid leads to the formation of amplicon replication compartments (ARCs) visualized by the TetR-EYFP fusion protein (Figure 4b). We then utilized these ARCs for FRAP experiments in order to estimate the diffusion kinetics of the amplicon DNA within ARCs. Interestingly, the fluorescence recovery of the bleached TetR-EYFP area within ARCs was much slower compared to the recovery dynamics of ICP4 in VRCs, reaching a maximum of approximately ~35% after 3 min, and the mean T-half was significantly prolonged (Figure 3b–d and Movie S6).

In summary, these results argue towards the possibility that HSV-1 VRCs have properties of phase-separated condensates.

To corroborate the hypothesis that HSV-1 VRCs are liquid–liquid phase-separated condensates, we used 3D live-cell imaging to assess three distinct properties of phase-separated condensates: the ability to fuse upon contact, to grow over time, and to maintain a spherical shape. Vero cells were infected with rHSV-EYFPICP4 and subjected to 3D live-cell imaging 6 h post infection (hpi) (Figure 4e). The infected cells were monitored for 3 h and z-stacks were captured every 10 min. The obtained datasets were processed for 3D re-modeling of representative VRCs and analyzed using Cell Profiler (Figure 4e–g). This 3D live-cell imaging setup allowed us to visualize the spatio-temporal dynamics of HSV-1 VRCs in real time and to quantify their liquid-like properties. We found that HSV-1 VRCs freely moved in the nuclei and coalesced upon close contact, confirming our previous findings (Figure 4e and Movie S7). The number of VRCs per nucleus significantly decreased during the course of infection (6–9 hpi) while the area of individual VRCs increased (Figure 4e–g). This suggests that individual VRCs merged (VRC fusion) and formed bigger inclusions (VRC growth) over time. However, unlike factories of RNA viruses, HSV-1 VRCs did not maintain a spherical shape after fusing and the compactness of VRCs markedly increased over time (Figure 4g).

Taken together, the 3D live-cell imaging data of infected cells revealed that HSV-1 VRCs share spatio-temporal properties of viral factories and cellular condensates, suggesting that they occur by phase separation.

## 3. Discussion

Compartments in cells can form by phase separation, as originally observed in C. elegans germline P granules by Brangwynne et al. [33]. The concept of LLPS has been extended to other organelles, such as the nucleolus, promyelocytic leukemia protein nuclear-bodies (PML NBs), stress granules (SGs), or Cajal bodies, to name a few (reviewed in [8,34,35,36]). Cell biological processes are driven by the de-mixing of macromolecules and nucleic acids at distinct sites, such as transcriptional hubs, splicing foci, or DNA repair loci [37,38,39,40,41,42]. Recently, it became clear that membrane-less viral factories in the cytoplasm that are formed by RNA viruses, such as VSV or RabV, share similarities with phase-separated compartments [6,7]. In particular, the spatio-temporal behavior of viral factories and the fact that they are formed by de-mixing of distinct vIDPs suggest that these assemblies are phase-separated condensates.

DNA viruses, including HSV-1, replicate in nuclear membrane-less VRCs with properties of viral factories and cellular condensates. It has been proposed recently in a handful of studies that HSV-1 VRCs might be phase-separated condensates as well [2,11] and that many HSV-1 proteins are vIDPs, such as the IE proteins (e.g., ICP4 or ICP27) and many L proteins (e.g., UL11) [13,43].

Here, we demonstrated that the essential viral TF ICP4 is a vIDP that can drive the formation of condensates in the nucleus via LLPS. Live-cell imaging and FRAP data indicated that ICP4 condensates share spatio-temporal properties with phase-separated condensates. Furthermore, we identified the ICP4 protein domains that drive LLPS (e.g., CTA) and also found that the NTA or the DBD control the liquid-like behavior of ICP4. Essentially, FRAP experiments showed that ICP4 retains its liquid-like nature also in HSV-1 VRCs, suggesting that they share this biophysical property with ICP4 condensates. A careful interrogation of the liquid-like characteristics of VRCs revealed that those membrane-less nuclear inclusions have many other liquid-like properties, such as the capability to fuse and grow over time. Our results corroborate the hypothesis that HSV-1 VRCs are liquid–liquid phase-separated condensates akin to viroplasms of RNA viruses.

Interestingly, evidence that fusion of HSV-1 VRCs might be an event that underlies liquid-like dynamics was reported almost ten years ago. Chang et al. found that HSV-1 VRCs freely move within the nucleus and coalesce particularly at nuclear speckles [12]. These membrane-less organelles (MLOs) are hubs for mRNA processing and, more importantly, they are phase-separated condensates (reviewed in [44,45]). Although convincing arguments from earlier studies and the results presented in this work together suggest that HSV-1 VRCs are phase-separated condensates, the biophysical properties of these viral assemblies do not fully comply with the liquid-like nature of cellular condensates. First, HSV-1 VRCs do not relax into spherical inclusions after fusion (see Figure 4g). This observation is in accordance with previous studies that found that the genomic content does not freely diffuse among fusing VRCs but rather maintains the original territory also at the fusion site [11,15]. It has been demonstrated that small areas at such fusion sites are the locations where vDNA recombination occurs, a process that is very typical for herpesviruses and a driving force for viral evolution [15]. In this respect, we found that the genomic content of ARCs also did not follow typical liquid-like FRAP dynamics, suggesting that the DNA within VRCs and ARCs acts as a scaffolding molecule rather than a driver of LLPS (see Figure 4a–d). Second, McSwiggen et al. described that the motion dynamics of the cellular RNA polymerase II (RNA-PolII) within HSV-1 VRCs do not follow liquid-like diffusion kinetics and concluded that HSV-1 VRCs may not be phase-separated condensates [13]. However, given the possibility that the HSV-1 DNA acts as a spatial scaffold rather than a phase-separating molecule, and that the RNA-PolII C-terminal domain (CTD) can undergo phase separation [38], we suggest that HSV-1 VRCs are liquid-like condensates that are likely formed, at least in part, via LLPS. However, whether ICP4 phase separation is the only driving force for the formation and maintenance of HSV-1 VRCs remains to be investigated.

Co-activator and TF condensation is a process required for gene control and is driven by their intrinsically disordered activation domains [37]. The formation of phase-separated ICP4 droplets in transfected cells is likely driven by the CTA or the linker region between the CTA and the DBD, whereas the NTA and the DBD are likely involved in controlling the protein phase behavior (see Figure 3). It has been shown by Dremel et al. that early in infection, ICP4 can bind to well-defined consensus sequences present at certain transcriptional start sites (TSS) on the host cell genome, likely via its DBD [46]. This explains, on one hand, why we did not observe any dots in addition to droplets with the ICP4 construct that lacks the NTA and the DBD. On the other hand, it reveals why we did not find any dots or droplets with the ICP4 construct that lacks the CTA and the DBD as this construct lost both capabilities, DNA binding and phase separation.

The notion that HSV-1 VRCs likely are phase-separated condensates and that the viral TF ICP4 is an IDP driving LLPS may have an impact on biomedical and molecular applications. On one hand, ICP4 can be utilized to study TF condensation in live cells without altering cellular genes and cell homeostasis. In addition, HSV-1 VRCs provide an accessible model system to study the liquid-like nature of phase-separated condensates in live cells. Furthermore, the majority of DNA viruses, such as adenoviruses, parvoviruses, papillomaviruses, or polyomaviruses, form nuclear VRCs in infected cells and the concept of LLPS may apply for the VRCs of these viruses as well (reviewed in [1,2,3,4]). By further exploring the molecular mechanisms behind how phase-separated VRCs are formed and maintained within the nucleus, we may also better understand viral replication processes and diseases.

## 4. Materials and Methods

### 4.1. Cells and Viruses

African green monkey kidney cells (Vero, ATCC) were maintained in Dulbecco’s modified Eagle medium (DMEM, Gibco, Fisher Scientific AG, Reinach, Switzerland, Cat. No. 31885-023) supplemented with 10% fetal bovine serum (FBS, AMIMED, BioConcept, Allschwil, Switzerland, Cat. No. 2-01F30-I) and 1% Antibiotic–Antimycotic (Streptomycin, Amphotericin B, Penicillin), Gibco, Fisher Scientific AG, Reinach, Switzerland, Cat. No. 15240-062). The cells were kept at 37 °C and 5% CO_2_ and passaged every 3–4 days at a confluency of <80% using 0.05% Trypsin–EDTA (Gibco, Fisher Scientific AG, Reinach, Switzerland, Cat. No. 25300-054). The recombinant HSV-1 expressing ICP4 which is N-terminally fused with the enhanced yellow fluorescent protein (rHSV-EYFPICP4) was a kind gift from Roger D. Everett and is described elsewhere [20]. The rHSV-EYFPICP4 and wt HSV-1 (F-strain) virus stocks were produced and titrated in Vero cells. Briefly, Vero cells were grown in a 75 cm^2^ cell culture flask at a confluency of 90% and then infected with rHSV-EYFPICP4 or wt HSV-1 at a MOI of 0.01 in 10 mL DMEM with 0% FBS and 0% Antibiotic–Antimycotic. The cells were then incubated at 4 °C for 30 min to establish virus attachment, followed by an incubation at 37 °C and 5% CO_2_ for 2 h. The infection medium was then replaced with DMEM supplemented with 2% FBS and 1% Antibiotic–Antimycotic and incubated at 37 °C and 5% CO_2_ for 4–5 days until the cytopathic effect (CPE) reached >90%. The supernatant was then removed and stored at 4 °C while the cells were harvested, pelleted, and subjected to three freezing and thawing cycles. The cell pellet was combined with the previously harvested supernatant and the virus stock was centrifuged for 10 min at 1900× *g* and kept at 4 °C. The cleared lysate was aliquoted and stored at −80 °C. The virus stocks were titrated by plaque assay as described elsewhere [47,48].

### 4.2. Plasmid and Amplicon Constructs

The plasmid expressing ICP4 that is N-terminally fused to EYFP (pIE3-EYFP-ICP4) was a kind gift from Roger D. Everett and is described elsewhere [20]. This plasmid was used to generate the EYFP control plasmid (pIE3-EYFP) expressing EYFP alone as follows: The ICP4 gene sequence was cut from the vector at the *Hin*dIII and *Kpn*I restriction sites and replaced with a duplex DNA oligomer (5′-gct caa gct tcg aat tct gca gtc gac taa cct cta gag gat ccc cgg gta ccg cgg-3′) that was cut with *Hin*dIII and *Kpn*I. The same pIE3-EYFPICP4 plasmid was used to generate the EYFP-ICP4 mutant constructs pIE3-EYFP-ICP4∆NTA, pIE3-EYFP-ICP4∆NTA∆DBD, and pIE3-EYFP-ICP4-NTAnls. pIE3-EYFP-ICP4∆NTA was cloned by cutting the NTA from the plasmid using *Eco*NI and *Xho*I restriction sites. The resulting gap in the plasmid was sealed by ligating a duplex DNA strand with corresponding restriction site overhangs (5′-ctc gag ctc aag ctt cga att ctg cag tcg aca tgc ccc tct ccg agg-3′). pIE3-EYFP-ICP4∆NTA∆DBD was cloned by inserting the duplex DNA (5′-ctc gag ctc aag ctt cga att ctg cag tcg aca tgc aga agg gct tc-3′) between the *Xho*I and *Xmn*I sites of pIE3-EYFP-ICP4. pIE3-EYFP-ICP4-NTAnls was generated by inserting the duplex DNA (5′-cct ctc cga ggc cgc gcc caa gcc ccg ggc ggc ggc gag gac ccg gga ggg gcg caa gcg caa gag tta agg cgc ccc cgg agg gtt tgg atc ctc tag agg atc ccc ggg tac c-3′) between the *Eco*NI and *Kpn*I sites of pIE3-EYFP-ICP4. All inserts were generated by annealing the corresponding ssDNA strands that were designed in order to have necessary restriction site overhangs already present. The ssDNA oligos were acquired from Microsynth (Balgach, Switzerland). The plasmid expressing ICP27 that is C-terminally fused to mCherry (mCherry2-ICP27) was a kind gift from Lucas Pelkmans and was constructed as follows: the ICP27 gene sequence was amplified from the plasmid pEBHICP27 [49] with the primers (5′-agc gaa ttc cgc cac cat ggc gac tga cat tga tat gct aat tg-3′) and (5′-gct ggt acc cca aac agg gag ttg caa taa aaa tat ttg ccg-3′) and the resulting ICP27 gene sequence was cloned between the *Eco*RI and *Kpn*I sites of mCherry2-N1 (Addgene Plasmid #54517). The plasmid pCI-mCherry expressing mCherry alone was a kind gift from Catherine Eichwald and is described elsewhere [50]. The TetO-TetR-based HSV-1 amplicon system that comprises the amplicon plasmid pHSV-TetO and the reporter plasmid pSV2-TetR-EYFP has been utilized in several studies in the past and is described elsewhere [30,31,51,52].

### 4.3. 3D Live-Cell Imaging

For the imaging of EYFPICP4 droplets, 1.5 × 10^5^ Vero cells were seeded in a 2-well microslide with a 0.17 mm glass bottom (µ-slide, 2 wells, ibidi, Vitaris AG, Baar, Switzerland, Cat.No. 80287) and incubated overnight. The next day, cells were transfected with 0.3 µg of pIE3-EYFPICP4 or pIE3-EYFP using Lipofectamine™ LTX and PLUS™ reagents (Invitrogen, Thermo Fisher Scientific, Zurich, Switzerland, Cat.No. 15338-100) according to the manufacturer’s instructions. The next day, cell nuclei were counterstained with Hoechst 33342 and subjected to 3D live-cell imaging using a spinning disc confocal microscope (ImageXpress Micro Confocal (IXM-C), Molecular Devices, LLC., San Jose, CA, USA) equipped with a 40x objective (NA 0.95). For the imaging of rHSV-EYFPICP4 viral replication compartments (VRCs), 1.5 × 10^5^ Vero cells were seeded in a 2-well microslide with a 0.17 mm glass bottom (µ-slide, 2 wells, ibidi, Vitaris AG, Baar, Switzerland, Cat.No. 80287) and incubated overnight. The next day, cells were infected with rHSV-EYFPICP4 at a MOI of 1. At 6 hpi, the nuclei were counterstained with Hoechst 33342 (Invitrogen, Thermo Fisher Scientific, Zurich, Switzerland, Cat.No. H3570) according to the manufacturer’s instructions. For live-cell imaging, the cell medium was replaced with 1 mL FluoroBrite DMEM (Gibco, Fisher Scientific AG, Reinach, Switzerland, Cat.No. A18967-01) supplemented with 2% FBS. For both transfection and infection experiments, the DAPI channel was acquired for Hoechst-stained nuclei and the GFP/FITC channel was acquired for the EYFPICP4 fusion proteins. Cells were imaged for 3 h and 50 z-stacks were acquired for the GFP/FITC channel at 10 min intervals, whereas only a single confocal image was acquired for the DAPI channel in order to reduce phototoxicity. At the beginning and the end of each live-cell imaging experiment, 50 z-stacks were taken for both channels (DAPI and GFP/FITC). The resulting datasets were used to calculate maximum-intensity projections (MIPs) of z-stacks and background-subtracted 3D reconstruction/rendering of nuclei and VRCs using Imaris v9.5 (Bitplane, Oxford Instruments, Bitplane AG, Zurich, Switzerland). Spatio-temporal analysis of VRCs was performed using customized Cell Profiler (v3.1.8) pipelines [53].

### 4.4. ICP4 Live-Cell Phase Diagrams

The EYFP-ICP4 live-cell phase diagrams were generated using a confocal laser scanning microscope (SP8, Leica, Leica Microsystems, Heerbrugg, Switzerland) equipped with a 63x objective (NA 1.4). Briefly, 1.2 × 10^5^ Vero cells were seeded in a 24-well tissue culture plate with a 0.17 mm glass bottom (Cellvis, Mountain View, CA, USA, Cat.No. P24-1.5H-N) and incubated overnight. The next day, different amounts (0.01, 0.05, 0.1, 0.15, 0.2, or 0.25 µg) of pIE3-EYFPICP4 or ICP27-mCherry constructs were transfected in biological triplicates using Lipofectamine™ LTX and PLUS™ reagents (Invitrogen, Thermo Fisher Scientific, Zurich, Switzerland, Cat.No. 15338-100) as described above. The cells were counterstained with Hoechst 33342 and subjected to live-cell imaging at 16 hpt. Randomly, 50–100 transfected cell nuclei from each biological triplicate were counted and categorized according to their fluorescence pattern: dots, droplets, or no pattern, where dots were defined as puncta with a diameter of <1 µm and droplets with a diameter of >1 µm. The nuclei where at least one droplet was found were considered as positive for droplets. The resulting datasets for all constructs were plotted and used to calculate second-order polynomial regression curves. The ICP4 regression curve for droplets was interpolated into a live-cell phase diagram using Prism 9 (GraphPad, San Diego, CA, USA) with the following equation:

Q = 1973 × [DNA]^2^ + 455.2 × [DNA] + 50.58

where Q is the fraction of transfected cells comprising ICP4 droplets, and [DNA] is the concentration of transfected DNA in µg.

Representative single z-scans were acquired from cells transfected with either EYFP-ICP4 or ICP27-mCherry constructs. The DAPI channel was acquired for Hoechst-stained nuclei, the EYFP channel was acquired for the EYFPICP4 fusion or EYFP proteins, and the mCherry channel was acquired for the ICP27mCherry fusion or mCherry proteins.

### 4.5. 1,6-Hexanediol Treatment

ICP4 droplet formation was induced in Vero cells as described above (see Section 4.4. ICP4 Live-Cell Phase Diagrams). Briefly, 0.25 µg of pIE3-EYFPICP4 plasmid DNA was transfected in Vero cells and at 24 hpt, the cells were counterstained with Hoechst 33342 and treated with 3.5% 1,6-Hexanediol (Merck, Schaffhausen, Switzerland, Cat.No. 8.04308.0100) in FluoroBrite DMEM (Gibco, Fisher Scientific AG, Reinach, Switzerland, Cat.No. A18967-01) supplemented with 2% FBS for 15 min. Fluorescence images of the blue (Hoechst 33342) and yellow (EYFP-ICP4) channels were acquired at indicated times using a SP8 CLSM (Leica, Leica Microsystems, Heerbrugg, Switzerland) with a 63x objective (NA 1.4). The cells were kept at constant conditions of 37 °C and 5% CO_2_ throughout the experiment.

### 4.6. FRAP Experiments

Fluorescence recovery after photobleaching (FRAP) experiments on rHSV-EYFPICP4 VRCs and pHSV-TetO amplicon replication compartments (ARCs) was performed on a SP8 CLSM (Leica, Leica Microsystems, Heerbrugg, Switzerland) with a 63x objective (NA 1.4). The FRAP experiments on EYFPICP4 droplets were performed using the SP8 Falcon CLSM (Leica, Leica Microsystems, Heerbrugg, Switzerland) equipped with a 63x objective (NA 1.4). The generation of rHSV-EYFPICP4 VRCs was achieved as described above. Briefly, Vero cells were infected with rHSV-EYFPICP4 at a MOI of 1, and at 12 hpi, the cells were counterstained with Hoechst 33342 and subjected to FRAP analysis. The generation of pHSV-TetO ARCs was achieved as follows: 1.5 × 10^5^ Vero cells were seeded in a 2-well microslide with a 0.17 mm glass bottom (µ-slide, 2 wells, ibidi, Vitaris AG, Baar, Switzerland, Cat.No. 80287) and incubated overnight. The next day, 0.2 µg of pHSV-TetO and 0.02 µg of pSV2-TetREYFP were transfected using Lipofectamine™ LTX and PLUS™ reagents (Invitrogen, Thermo Fisher Scientific, Zurich, Switzerland, Cat.No. 15338-100) as described above. At 16 hpt, the cells were infected with wt HSV-1 (F-strain) at a MOI of 2. The cells were screened for the development of ARCs starting at 12 hpi and were counterstained with Hoechst 33342 for FRAP analysis at approx. 20 hpi. EYFPICP4 droplets were generated by transfecting Vero cells with 0.15 µg of pIE3-EYFPICP4 as described above and subjected to FRAP analysis at 24 hpt. All FRAP experiments were conducted in FluoroBrite DMEM (Gibco, Fisher Scientific AG, Reinach, Switzerland, Cat.No. A18967-01). FRAP experiments were performed using the FRAP function of the LasX software (Leica) as follows: a circular area of 1 µm in diameter was bleached by applying 10 iterations with the 405 nm laser at 100% laser power. The fluorescent recovery was monitored by taking fluorescence images of the EYFP channel at a 1 s interval for 3 min. For each FRAP dataset, a circular area of 1 µm was used as the fluorescent control and a squared area of 5 × 5 µm was chosen as background. The resulting FRAP datasets were analyzed using the easyFRAP-web interface [54]. Fully normalized data were used to generate FRAP diagrams and calculate recovery half-times (T-half) from 6–12 independent measurements. Representative images were taken and processed for each FRAP experiment using the Imaris software v9.5 (Bitplane, Oxford Instruments, Bitplane AG, Zurich, Switzerland). Fluorescent intensities of FRAP movies were normalized using a customized Fiji pipeline [55].

### 4.7. ATP Depletion

Depletion of cellular ATP levels was accomplished by culturing the cells with glucose-free DMEM (Gibco, Fisher Scientific AG, Reinach, Switzerland, Cat.No. 11966-025) for 2 h followed by a treatment with 500 nM Oligomycin A (Tocris Bioscience, Bio-Techne, Minneapolis, MN, USA, Cat.No. 4110) for 30 min. The optimal Oligomycin A concentration was determined empirically and tested using a commercially available ATP detection system (CellTiter-Glo^®^ Luminescent Cell Viability Assay, Promega, Dübendorf, Switzerland, Cat.No. G7570) according to the manufacturer’s instructions.

### 4.8. Western Blot Analysis

To confirm the molecular size and estimate the different expression levels of the mutant and wt EYFP-ICP4 constructs, a Western blot analysis was performed as follows: The day before infection, 1.2 × 10^5^ Vero cells/well were seeded in 24-well plates. Transfections were carried out as described for 3D live-cell imaging. At 24 hpi, the cells were washed with PBS and lysed in 100 µL protein loading buffer (62 mM Tris base, 2% sodium dodecyl sulfate (SDS), 5% 2-mercaptoethanol, 10% glycerol, and 0.005% bromphenol blue). The resulting cell lysates were boiled for 10 min and stored at −20 °C for further analysis. A total of 20 µL lysates per sample were separated in an 8% SDS–polyacrylamide gel and transferred to nitrocellulose membranes (Whatman, Fisher Scientific AG, Reinach, Switzerland). The membranes were then blocked with 5% nonfat dry milk in PBS-T (PBS supplemented with 0.3% Tween 20) for 1 h at RT. Incubation with antibodies was carried out in PBS-T supplemented with 2.5% dry milk. The following primary antibodies were used: for the detection of EYFP-ICP4, we used the ms mAb to HSV-1 ICP4 (10F1) from Abcam (Cat.No. ab6514) at a dilution of 1:500. To detect the mutant EYFP-ICP4 constructs, we used the ms mAb to GFP (JL-8) from Clontech (Clontech, Takara Bio Europe SAS, Saint-Germain-en-Laye, France, Cat.No. 632380) at a concentration of 1:10,000, and finally, for b-Actin, we used the ms mAb to b-Actin from Sigma (Merck, Schaffhausen, Switzerland, Cat.No. A2228) at a concentration of 1:10,000. In order to detect the primary antibodies, we used the gt x ms IRDye 800CW antibody from Licor (LI-COR Biosciences GmbH, Bad Homburg, Germany, Cat.No. 926-32210) at a concentration of 1:5000. The secondary antibodies were visualized using an Odyssey^®^ Fc Imaging System from Licor (LI-COR Biosciences GmbH, Bad Homburg, Germany).

### 4.9. Prediction Algorithms

The total percentage disorder scores of each protein analyzed in this study was calculated using the various, short, and long 2 (VSL2) algorithm from the predictor of natural disordered regions (PONDR) platform (Molecular Kinetics, La Pas Trail, Indianapolis, IN, USA, www.pondr.com (accessed on October 1st 2020)). The net charge per residue (NCPR) scores/graphics were obtained from the online tool Classification of Intrinsically Disordered Ensemble Regions (CIDER, [56]). The pi–pi interaction prediction was performed using the python script from Vernon et al. [57]. All raw output data were processed with Prism 9 (GraphPad, San Diego, CA, USA).

### 4.10. Statistical Analysis

Statistical significance was assessed for all datasets using an unpaired *t*-test with Welch’s correction. All statistical analysis was performed using Prism 9 (GraphPad, San Diego, CA, USA).

## Data Availability

The data presented in this study are available on request from the corresponding author. The data are not publicly available due to internet access restrictions of our data servers.

## Supplementary Materials

Supplementary materials can be found at https://www.mdpi.com/article/10.3390/ijms22094447/s1.
