# Supplementary material for: The HSV-1 Transcription Factor ICP4 Confers Liquid-Like Properties to Viral Replication Compartments
Source: Int J Mol Sci. 2021 Apr 24;22(9):4447. doi: 10.3390/ijms22094447 (PMC8123221; doi:10.3390/ijms22094447)
Supplement: Supplementary file 1 [file ijms-22-04447-s001.zip › ijms-1150917-supplementary-4.29.pdf]

International Journal of
Molecular Sciences

Table S1. Examples of cited viral IDPs that can undergo LLPS

#	Accession #	Protein Name	VSL2 score [%]	Citation
1	Q8AZK7	EBV EBNALP	100.0	[1]
2	P12978	EBV EBNA2	91.2	[1]
3	Q77M42	MeV Phosphoprotein (P)	77.5	[2,3]
4	P03520	VSV Phosphoprotein (P)	59.6	[4,5]
5	P16286	RABV Phosphoprotein (P)	49.5	[6]
6	P03466	IVA Nucleoprotein (NP)	46.6	[7]
7	Q77M43	MeV Nucleoprotein (N)	38.3	[2,3]
8	P03433	IVA (PA)	35.6	[7]
9	P16285	RABV Nucleoprotein (N)	25.1	[6]
10	P03523	VSV RdR Pol (L)	19.0	[4]
11	P03521	VSV Nucleoprotein (N)	17.3	[4]

The overall percentage VSL2 scores (VSL2 score [%]) and the corresponding citations are annotated. The viral proteins at the bottom of the list were not considered because they are either not able to undergo LLPS per se, or they are auxiliary factors promoting LLPS of other viral proteins (grey box).

Table S2. HSV-1 proteome disorder scores

#	Gene/ORF	Accession #	Protein Name	VSL2 score [%]	e	ne	IE	E	L	ns	cap	teg	env
1	*US11*	P04487	Accessory factor, RNA binding protein US11	100.0									
2	*γ1 34.5 / RL1*	P36313	Neurovirulence factor ICP34.5	100.0									
3	*UL56*	P10240	Protein UL56	95.9									
4	*US12*	P03170	ICP47 protein	81.8									
5	*US1*	P04485	Transcriptional regulator ICP22	78.6									
6	*UL49*	P10233	Tegument protein VP22	78.1									
7	*α4*	P08392	Major viral transcription factor ICP4	74.9									
8	*α0 / RL2*	P08393	(IE110) E3 ubiquitin -protein ligase	74.8									
9	*US4*	P06484	Envelope glycoprotein G	71.9									
10	*UL14*	P04291	Tegument protein UL14	70.3									
11	*UL26*	P10210	Capsid scaffolding protein	63.9									
12	*UL11*	P04289	Cytoplasmic envelopment protein 3	63.5									
13	*US10*	P06486	Viral protein US10	61.9									
14	*UL3*	P10187	Nuclear phosphoprotein UL3	60.4									
15	*UL54*	P10238	RNA processing protein ICP27	57.0									
16	*US9*	P06481	Envelope protein US9	56.7									
17	*UL36*	P10220	Large tegument protein deneddylase	53.2									
18	*US8*	P04488	Envelope glycoprotein E	49.5									
19	*US3*	P04413	Serine/threonine-protein kinase US3	48.4									
20	*UL48*	P06492	Tegument protein VP16	47.6									
21	*UL46*	P10230	Tegument protein UL46, VP11/12	46.8									
22	*UL47*	P10231	Tegument protein UL47, VP13/14	46.5									
23	*US7*	P06487	Envelope glycoprotein I	46.4									
24	*UL42*	P10226	DNA polymerase processivity factor	45.7									
25	*US2*	P06485	Protein US2	43.0									
26	*UL24*	P10208	Protein UL24	42.8									
27	*UL38*	P02310	Triplex capsid protein VP19C	42.7									
28	*UL12*	P04294	Alkaline nuclease	42.7									
29	*UL32*	P10216	Packaging protein	40.8									
30	*UL44*	P10228	Envelope glycoprotein C	40.3									
31	*UL34*	P10218	Nuclear egress protein 2	39.3									
32	*UL51*	P10235	Tegument protein UL51	38.9									
33	*UL35*	P10219	Small capsomere-interacting protein, VP26	38.4									
34	*UL2*	P10186	Uracil-DNA glycosylase	38.0									
35	*UL1*	P10185	Glycoprotein L	37.5									
36	*UL41*	P10225	Virion host shutoff protein	37.2									
37	*UL33*	P10217	Tripartite terminase subunit 2	33.9									
38	*UL4*	P10188	Nuclear protein UL4	33.2									
39	*US5*	P06480	Envelope glycoprotein J	32.6									
40	*UL27*	P10211	Glycoprotein B	31.2									
41	*UL39*	P08543	Ribonucleoside-diphosphate reductase large subunit	30.5									
42	*UL6*	P10190	Portal protein	29.7									
43	*UL43*	P10227	Membrane protein UL43	29.5									
44	*UL37*	P10221	Inner tegument protein	28.9									
45	*UL25*	P10209	Capsid vertex component 2	28.6									
46	*UL13*	P04290	Serine/threonine-protein kinase UL13	28.6									
47	*UL17*	P10201	Capsid vertex component 1	28.0									
48	*UL55*	P10239	Tegument protein UL55	28.0									
49	*UL10*	P04288	Glycoprotein M	27.3									
50	*UL16*	P10200	Cytoplasmic envelopment protein 2	27.1									
51	*UL50*	P10234	Deoxyuridine 5'-triphosphate nucleotidohydrolase	26.7									
52	*UL23*	P03176	Thymidine kinase	26.1									

53	*UL21*	P10205	Tegument protein UL21	25.8
54	*UL22*	P06477	Glycoprotein H	25.5
55	*US6*	Q69091	Envelope glycoprotein D	25.4
56	*UL28*	P10212	Tripartite terminase subunit 1	25.2
57	*UL52*	P10236	DNA primase	24.5
58	*UL31*	P10215	Nuclear egress protein 1	22.9
59	*UL20*	P10204	Protein UL20	22.1
60	*UL29*	P04296	Major DNA-binding protein ICP8	21.7
61	*UL30*	P04293	DNA polymerase catalytic subunit	21.5
62	*UL40*	P10224 / F8RDD9	Ribonucleoside-diphosphate reductase small subunit	18.3
63	*UL5*	P10189 / Q2MGV2	DNA replication helicase	17.9
64	*UL45*	P10229	Envelope protein UL45	17.4
65	*UL15*	P04295	Tripartite terminase subunit 3	15.9
66	*UL9*	P10193	Replication origin-binding protein	14.7
67	*UL7*	P10191	Virion tegument protein	12.8
68	*UL18*	P10202	Triplex capsid protein 2, VP23	12.0
69	*UL19*	P06491 / G8HBD2	Major capsid protein, VP5	11.6
70	*UL53*	P68331	Glycoprotein K	9.5
71	*UL8*	P10192	DNA helicase/primase complex-associated protein	4.8
72	*ORF-P*	n/a	ORF-P protein	n/a
73	*ORF-O*	n/a	ORF-O protein	n/a
74	*LRP1*	P17588	Latency-related transcript 1	n/a
75	*LRP2*	P17589	Latency-related transcript 2	n/a

A total of 71 proteins of the HSV-1 proteome were assessed with the VSL2 algorithm of the online platform PONDR. The following features are annotated for each protein: Overall percentage VSL2 scores (VSL2 score [%]), if it is essential (e) or non-essential (ne), if it is an immediate early (IE), early (E) or late (L) protein, and if it is non-structural (ns), a capsid (cap), a tegument (teg) or an envelope (env) protein. The four latency-associated proteins/transcripts at the bottom of the list were not considered (grey box). Green boxes indicate that the protein belongs to the corresponding protein class in the column; yellow boxes = not essential in non-dividing cells. The essential HSV-1 IE proteins ICP4 and ICP27 are highlighted in red.

Table S3. Examples of cited human IDPs that can undergo LLPS

#	Accession #	Protein Name	VSL2 score [%]	Citation
1	Q01844	EWS	93.8	[8]
2	Q92804	Taf15	92.6	[8]
3	P35637	FUS	90.7	[8]
4	O60885	BRD4	86.9	[9]
5	Q01130	SRSF2	83.7	[10]
6	Q14103	hnRNPD	83.1	[11]
7	Q96PK6	RBM14	81.3	[10]
8	P08047	SP1	79.2	[8]
9	O14979	hnRDL	76.9	[11]
10	Q9UER7	DAXX	73.0	[12]
11	Q01860	OCT4	71.9	[13]
12	P09651	hnRNPA1	70.2	[10]
13	P51991	hnRNPA3	70.1	[11]
14	P22626	hnRNPA2B1	70.0	[11]
15	Q15648	MED1	70.0	[9]
16	P06748	NPM1	61.2	[12]
17	Q13148	TDP-43	57.3	[10]
18	P29590	PML	53.1	[14]
19	P22087	Fibrillarin	45.2	[10]
20	Q9NQI0	DDX4	45.0	[10]
21	P24928	RPB1	43.2	[15]
22	P31483	Tia1	38.6	[10]
23	P35222	β-catenin	30.4	[16]
24	P42858	Huntingtin	29.2	[14]

The overall percentage VSL2 scores (VSL2 score [%]) and the corresponding citations are annotated. This list is not exhaustive and represents only a selection of relevant human proteins that can undergo LLPS.

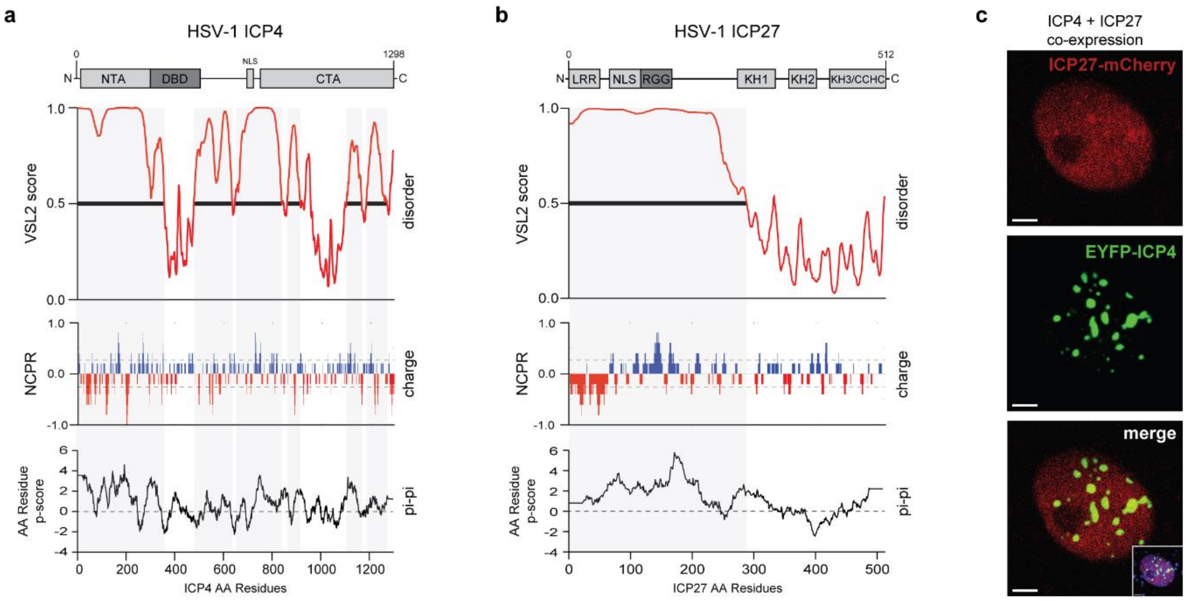

Figure S1. ICP4 and ICP27 LLPS predictors; The ICP4 (**a**) and ICP27 (**b**) relevant protein domains, the VSL2 disorder score profiles, net charge per residue (NCPR) and AA-residue p-scores of the pi-pi stacking predictor are shown. The IDRs of each protein are highlighted with a horizontal black line and a light-grey box. (**c**) ICP4 droplet formation is independent of ICP27. Vero cells were co-transfected with 0.1 µg of mCherry2-ICP27 and 0.15 µg of pIE3-EYFPICP4. The cells were counterstained with Hoechst 33342 at 16 hpt and subjected to CLSM. Scale bar: 3 µm;.

References

1. Peng, Q.; Wang, L.; Qin, Z.; Wang, J.; Zheng, X.; Wei, L.; Zhang, X.; Zhang, X.; Liu, C.; Li, Z., et al. Phase Separation of Epstein-Barr Virus EBNA2 and Its Coactivator EBNALP Controls Gene Expression. *J Virol* **2020**, *94*, doi:10.1128/JVI.01771-19.
2. Guseva, S.; Milles, S.; Jensen, M.R.; Salvi, N.; Kleman, J.P.; Maurin, D.; Ruigrok, R.W.H.; Blackledge, M. Measles virus nucleo- and phosphoproteins form liquid-like phase-separated compartments that promote nucleocapsid assembly. *Sci Adv* **2020**, *6*, eaaz7095, doi:10.1126/sciadv.aaz7095.
3. Zhou, Y.; Su, J.M.; Samuel, C.E.; Ma, D. Measles Virus Forms Inclusion Bodies with Properties of Liquid Organelles. *J Virol* **2019**, *93*, doi:10.1128/JVI.00948-19.
4. Heinrich, B.S.; Maliga, Z.; Stein, D.A.; Hyman, A.A.; Whelan, S.P.J. Phase Transitions Drive the Formation of Vesicular Stomatitis Virus Replication Compartments. *mBio* **2018**, *9*, doi:10.1128/mBio.02290-17.
5. Leyrat, C.; Schneider, R.; Ribeiro, E.A., Jr.; Yabukarski, F.; Yao, M.; Gerard, F.C.; Jensen, M.R.; Ruigrok, R.W.; Blackledge, M.; Jamin, M. Ensemble structure of the modular and flexible full-length vesicular stomatitis virus phosphoprotein. *J Mol Biol* **2012**, *423*, 182-197, doi:10.1016/j.jmb.2012.07.003.
6. Nikolic, J.; Le Bars, R.; Lama, Z.; Scrima, N.; Lagaudriere-Gesbert, C.; Gaudin, Y.; Blondel, D. Negri bodies are viral factories with properties of liquid organelles. *Nat Commun* **2017**, *8*, 58, doi:10.1038/s41467-017-00102-9.
7. Alenquer, M.; Vale-Costa, S.; Etibor, T.A.; Ferreira, F.; Sousa, A.L.; Amorim, M.J. Influenza A virus ribonucleoproteins form liquid organelles at endoplasmic reticulum exit sites. *Nat Commun* **2019**, *10*, 1629, doi:10.1038/s41467-019-09549-4.

8. Chong, S.; Dugast-Darzacq, C.; Liu, Z.; Dong, P.; Dailey, G.M.; Cattoglio, C.; Heckert, A.; Banala, S.; Lavis, L.; Darzacq, X., et al. Imaging dynamic and selective low-complexity domain interactions that control gene transcription. *Science* **2018**, *361*, doi:10.1126/science.aar2555.
9. Sabari, B.R.; Dall'Agnese, A.; Boija, A.; Klein, I.A.; Coffey, E.L.; Shrinivas, K.; Abraham, B.J.; Hannett, N.M.; Zamudio, A.V.; Manteiga, J.C., et al. Coactivator condensation at super-enhancers links phase separation and gene control. *Science* **2018**, *361*, doi:10.1126/science.aar3958.
10. Courchaine, E.M.; Lu, A.; Neugebauer, K.M. Droplet organelles? *EMBO J* **2016**, *35*, 1603-1612, doi:10.15252/embj.201593517.
11. Wang, J.; Choi, J.M.; Holehouse, A.S.; Lee, H.O.; Zhang, X.; Jahnel, M.; Maharana, S.; Lemaitre, R.; Pozniakovsky, A.; Drechsel, D., et al. A Molecular Grammar Governing the Driving Forces for Phase Separation of Prion-like RNA Binding Proteins. *Cell* **2018**, *174*, 688-699 e616, doi:10.1016/j.cell.2018.06.006.
12. Banani, S.F.; Lee, H.O.; Hyman, A.A.; Rosen, M.K. Biomolecular condensates: organizers of cellular biochemistry. *Nat Rev Mol Cell Biol* **2017**, *18*, 285-298, doi:10.1038/nrm.2017.7.
13. Boija, A.; Klein, I.A.; Sabari, B.R.; Dall'Agnese, A.; Coffey, E.L.; Zamudio, A.V.; Li, C.H.; Shrinivas, K.; Manteiga, J.C.; Hannett, N.M., et al. Transcription Factors Activate Genes through the Phase-Separation Capacity of Their Activation Domains. *Cell* **2018**, *175*, 1842-1855 e1816, doi:10.1016/j.cell.2018.10.042.
14. Bergeron-Sandoval, L.P.; Safaee, N.; Michnick, S.W. Mechanisms and Consequences of Macromolecular Phase Separation. *Cell* **2016**, *165*, 1067-1079, doi:10.1016/j.cell.2016.05.026.
15. Boehning, M.; Dugast-Darzacq, C.; Rankovic, M.; Hansen, A.S.; Yu, T.; Marie-Nelly, H.; McSwiggen, D.T.; Kokic, G.; Dailey, G.M.; Cramer, P., et al. RNA polymerase II clustering through carboxy-terminal domain phase separation. *Nat Struct Mol Biol* **2018**, *25*, 833-840, doi:10.1038/s41594-018-0112-y.
16. Zamudio, A.V.; Dall'Agnese, A.; Henninger, J.E.; Manteiga, J.C.; Afeyan, L.K.; Hannett, N.M.; Coffey, E.L.; Li, C.H.; Oksuz, O.; Sabari, B.R., et al. Mediator Condensates Localize Signaling Factors to Key Cell Identity Genes. *Mol Cell* **2019**, *76*, 753-766 e756, doi:10.1016/j.molcel.2019.08.016.

© 2021 by the authors. Submitted for possible open access publication under the terms and conditions of the Creative Commons Attribution (CC BY) license (http://creativecommons.org/licenses/by/4.0/).
